# Indiana Institute of Homœopathy

**Published:** 1892-09

**Authors:** 


### INDIANA INSTITUTE OF HOMŒOPATHY.

The Indiana State Society of Homœopathic Physicians held its twenty-sixth annual meeting Wednesday, May 18th. After the usual routine business and the reports of the Treasurer and Secretary, Dr. J. S. Martin, of Muncie, and Dr. W. B. Clarke, of Indianapolis, the President's address (Dr. J. T. Boyd), of Indianapolis, was delivered. He said that exactly forty years ago he delivered the President's address before the State Society of Ohio (old school), and then went on to show, in a long and able address, the improvement that has come in the practice of medicine by and through Homœopathy. He recommended that the Legislature of Indiana put one of the insane asylums under homœopathic charge, and also establish an inebriate asylum. Dr. G. W. Bowen, of Fort Wayne, read a paper on "Retained Placenta."

The first paper of the afternoon was one on "Sun Spots and Epidemics," by Dr. T. C. Duncan, of Chicago, President of the American Health Resort Association.

Dr. J. N. Taylor, President of the State Board of Health, followed with a paper entitled, "Prophylaxis, or Coincidence?" bringing up the question whether Belladonna is really a preventive of scarlatina, the trend of discussion going to show that it is. Dr. W. R. Bentley, of Morristown, read descriptions of three cases treated by him—one of hip-joint disease in a boy, another a chronic sore on the back of the hand of a farmer, the third a hemorrhage of the lungs in a child—all doing well.

Dr. F. C. Stewart, of Peru, read an interesting paper on "Microbes and the Chemical Changes They Cause," which was well discussed by Professors Buck and Fellows, Drs. Runnels, Waters, George, Stewart, Duncan, Taylor, Sawyer, and Clarke. Dr. J. D. George, of Indianapolis, read a paper on "La Grippe," recounting some of its more peculiar symptoms and how he met them. Dr. D. M. Bonham, of Edna Mills, read a short paper on "Intussusception of the Bowels," and was followed by Dr. W. B. Clarke, of Indianapolis, with an interesting paper entitled "Suicides in Indiana During 1891," giving statistics regarding four hundred and thirty cases, made up from a list kept up by him. This was freely discussed by Drs. Stewart, Fellows, Waters, and Huron. The feeling seemed to prevail that many cases of suicide resulted from the free use of "pick-me-ups" and the patent medicines with which this country is now being flooded.

In the evening Dr. F. H. Huron, of Danville, described a severe case of pneumonia in an infant—respiration 120 and pulse 220 a minute—which recovered under his care.

Professor J. D. Buck, of Cincinnati, called attention to some new and important books by Professors Crooks and Richardson, on the origin of the elements. Dr. J. W. Smith followed with a paper on "Some Hints on the Eyes," interesting and practical, after which Professor T. M. Stewart, of Cincinnati, an eye specialist, read a valuable paper on "The Eye and General Diagnosis."

The new members admitted were Drs. W. R. Stewart, of Wabash; W. B. Stewart, of Peru; G. W. Bernard, of Mulberry; Annie B. Campbell, Rockville; J. B. Westcott, Goodland; J. E. Wright, Cambridge City; Oran D. Thompson, Greensburg.

Dr. T. C. Duncan, of Chicago, President of the American

Health Resort Association, was then invited to speak regarding the measure introduced in Congress by Senator Gallinger to provide a national sanitarium for the treatment of pulmonary diseases, which he did entertainingly, and the Society passed a strong resolution indorsing the measure.

Dr. E. B. Grosvenor, of Richmond, recited two cases, one of malformed knees, the other of brain tumor in an infant, both doing well. He asked for some information regarding intussusception, and in the discussion the notable case of recent death in Indianapolis was referred to. Dr. W. T. Gott, of Crawfordsville, read an interesting paper, descriptive of the use of sponge as grafts for the production of new skin, for burns and injuries. Dr. H. B. Fellows, professor of mental and nervous diseases in Hahnemann College, Chicago, then gave a classical lecture on the "Physiology of the Action of the Heart and Lungs, especially in its Relation to Brain and Nervous Disease." Dr. G. W. Bowen read a paper on "Neurosis," and was emphatic in his belief that meat-eaters are not nervous, contrary to the popular belief. Dr. Buck, of Cincinnati, was called upon to speak to the question, and rather opposed the meat view, though he was very conservative. Drs. Taylor, Waters, and Clarke also spoke to the questions and propositions advanced by the speaker.

Dr. L. W. Jordan, an eye specialist, read descriptions of some interesting cases which had come under his observation. Dr. O. S. Runnels, followed with a fine essay on "The Vis Medicatrix Naturæ," covering the wide field of nutrition and life, and the problems of nature connected with them. Professor Buck, of Cincinnati, made a masterly philosophical address on the topics touched upon in Dr. Runnels's paper. Drs. Stewart and Bentley also spoke pertinently.

Dr. Alice C. Nivison, of Lafayette, then read the history of a complicated case, like neuralgia, asking advice, which was responded to by Drs. Runnels and Sawyer.

The election of officers then took place, resulting: President, M. H. Waters, M. D., Terre Haute; First Vice-President, W. T. Gott, M. D., Crawfordsville; Second Vice-President, E. B.

Grosvenor, M. D., Richmond; Treasurer, I. S. Martin, M. D., Muncie; Secretary, W. B. Clarke, M. D., Indianapolis.

In the afternoon, Dr. M. H. Waters, of Terre Haute, read a valuable paper on "Abominal Palpation," followed with one by Dr. L. W. Jordan, of Indianapolis, on the use of the hot needle in removing fleshy hypertrophies in the nose, and one by Dr. J. N. Taylor, of Crawfordsville, on "Mucous Polypus of the Nose." These papers stirred up a lively discussion as to the cause of these troubles. Dr. G. W. Bowen, of Fort Wayne, read a paper on "Materia Medica" that was carefully prepared, in which he claimed that many diseases are curable which are generally considered incurable. He was followed by D. W. Rowley, of Indianapolis, who detailed some cases in his practice.

A notable paper then followed, read by the Secretary, written by Dr. F. Kraft, of Cleveland, Ohio, editor of the *American Homœopathist*, entitled "Tic-douloureux," detailing an interesting case that had been through all kinds of treatment, and was finally cured easily.

A resolution was adopted directing the appointment of a committee to take steps to secure control for the homœopaths of one of the State hospitals for the insane. The discussion brought out a claim that the homœopaths in other States reduced the cost of medical treatment about one-half. It was decided to intrust the work of the former committee on this subject, with Dr. J. N. Taylor, Crawfordsville, Chairman, and with the new President, Dr. Waters, and Dr. Grosvenor, of Richmond, added.

"The Keeley Cure" came up for consideration and was freely discussed, a resolution being introduced and passed to the effect that no definite action could be taken by the Society until more was known about it. The Society then, after transacting routine business and thanking the press and the custodian of the State-house for favors shown, adjourned till next year.

